# Spinal Cord Ischemia Following Endovascular Abdominal Aortic Aneurysm Repair: An Unpredictable Catastrophe

**DOI:** 10.7759/cureus.35953

**Published:** 2023-03-09

**Authors:** Mohamed Ghoweba, Shaza Moussa, Oscar Chastain, Shafik Hanna-Moussa

**Affiliations:** 1 Internal Medicine, Texas A&M College of Medicine/CHRISTUS Good Shepherd Medical Center, Longview, USA; 2 Internal Medicine, Special Health Resources, Tyler Health Clinic, Tyler, USA; 3 Cardiothoracic Surgery, CHRISTUS Good Shepherd Medical Center, Longivew, USA; 4 Cardiology, Texas A&M College of Medicine/CHRISTUS Good Shepherd Medical Center, Longview, USA

**Keywords:** stent graft, endovascular complications, endovascular abdominal aortic aneurysm repair, neurologic injury, spinal cord ischemia

## Abstract

Spinal cord ischemia (SCI) following endovascular abdominal aortic aneurysm (AAA) repair (EVAR) is a rare yet catastrophic complication. The underlying pathophysiological mechanism remains incompletely understood. We present the case of a 75-year-old man with a difficult left common iliac artery (CIA) anatomy that necessitated the coiling of his left internal iliac artery (IIA) to ensure proper sealing of his aortic stent graft. The patient complained of bilateral lower extremity weakness immediately following the procedure. The patient was diagnosed with SCI, which was later confirmed by magnetic resonance imaging (MRI). He was treated with cerebrospinal fluid drainage. The patient's neurological status mildly improved on follow-up one year later.

## Introduction

Spinal cord ischemia (SCI) is a serious complication of endovascular abdominal aortic aneurysm (AAA) repair (EVAR). It remains underreported in the literature, and its pathophysiology is not fully elucidated. It has a reported occurrence of 0.21%. Early detection is essential in preventing long-term sequelae [1].

SCI symptoms mostly present immediately following the procedure; however, delayed SCI following EVAR has been reported in the literature [2-7]. A study showed that about a third of the patients with post-EVAR SCI had one internal iliac artery embolized by coils or covered with the stent graft, while about 14% had both internal iliac arteries compromised. Patients predominantly presented with paralysis or paraplegia, diminished sensations, as well as urinary and/or fecal incontinence. The physical and psychological sequelae of SCI are significant and are linked to decreased survival [8].

## Case presentation

**Figure 1:**
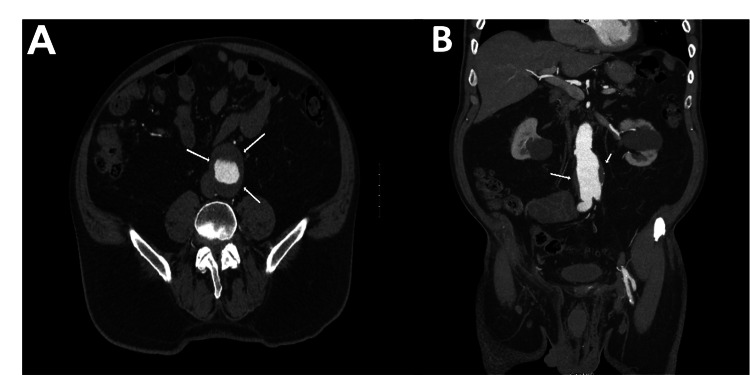
Abdominal aortic aneurysm: axial (panel A) and coronal (panel B) pre-procedural computed tomography angiograph slices showing the abdominal aortic aneurysm (white arrows).

**Figure 2:**
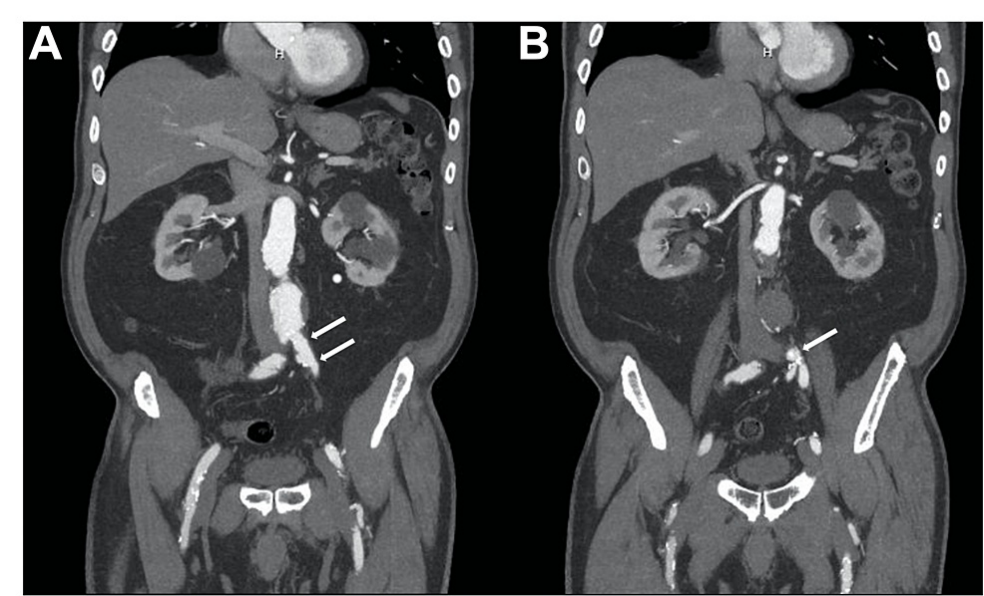
Short left common iliac artery: coronal pre-procedural computed tomography angiography coronal slices showing the left common iliac artery (panel A) and bifurcation (panel B). Note the short left common iliac artery of less than 1 cm in length (white arrows).

A 75-year-old man with a past medical history significant for AAA, chronic back pain, hypertension, non-insulin-dependent diabetes mellitus, chronic kidney disease, and hyperlipidemia presented to the outpatient cardiology clinic for follow-up of his AAA. A computed tomography angiography (CTA) abdomen/pelvis with and without contrast showed an increased aneurysm diameter of 5.5 cm with a length of 7 cm; thus, an EVAR was planned (Figure 1). A short left common iliac artery (CIA) with a length of 0.9 cm was noted, which would likely hinder a proper sealing zone for a stent graft. A decision was made to coil the left internal iliac artery (IIA) and extend the stent graft across the bifurcating sections into the left external iliac artery (EIA) (Figure 2).

**Figure 3:**
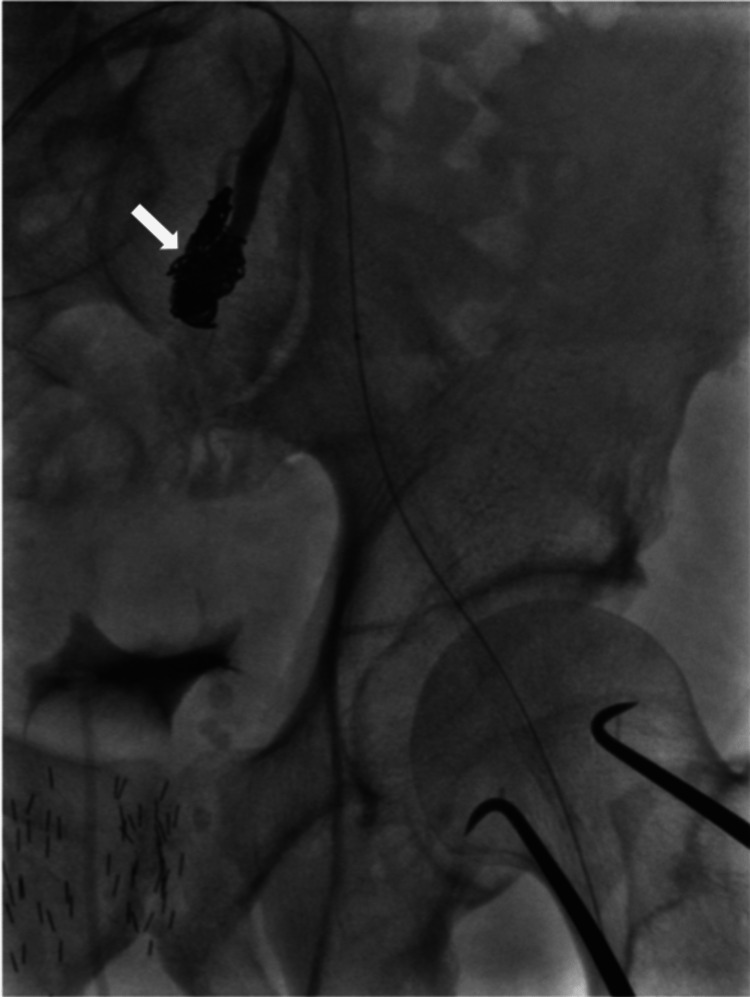
Coiling of the internal iliac artery: multiple cook coils were deployed over a catheter to occlude the proximal portion of the internal iliac artery (white arrow).

**Figure 4:**
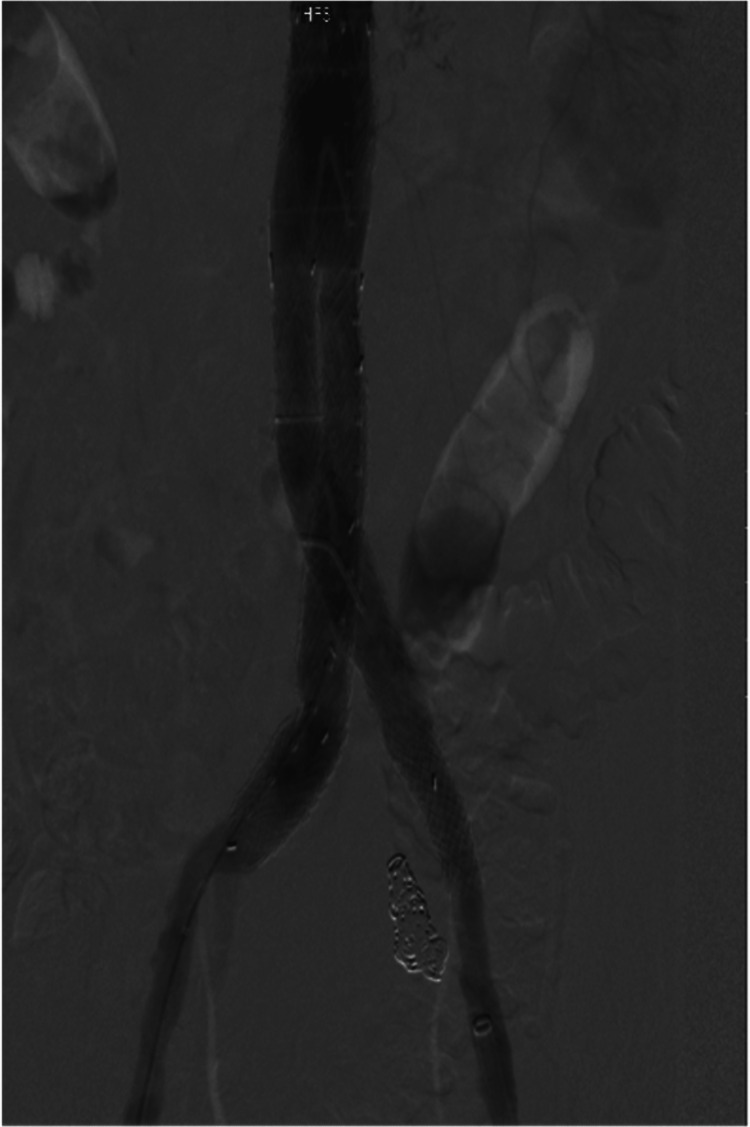
Regain of blood flow: completion angiogram showing the regain of blood flow.

During the procedure, an ultrasound-guided percutaneous approach was considered for the patient. However, given the patient's significant atherosclerotic disease, the surgical team opted against it. Therefore, a surgical cutdown to the common femoral arteries (CFA) was performed bilaterally. Sheaths were introduced into the right and left CFAs. A catheter was advanced up and over the left CIA. The origin of the left IIA was cannulated. Multiple Tornado (Cook Medical, Bloomington, Indiana, USA) embolization coils (8-0 and 7-0) were deployed over a catheter to occlude the proximal portion of the IIA (Figure 3). The femoral sheaths were exchanged for large 18- and 12-Fr DrySeal (Gore Medical, Flagstaff, Arizona, USA) sheaths over stiff wires. A distal aortic angiogram was performed using a pigtail catheter. The lowest renal artery was identified. The main body of a 31 cm × 14 cm × 13 cm EXCLUDER stent graft (Gore) was deployed. Cannulation of the contralateral limb was performed. An extension of the left contralateral limb was performed using a 14 cm × 12 cm limb extender (Gore). Following this, a 1.1 cm × 3.9 cm VIABAHN balloon-expandable VBX (Gore) stent was used as a further extension on the left contralateral limb. The ipsilateral limb was then extended using a 20 cm × 12 cm limb extender (Gore). Post-dilatation balloon angioplasty at all the connection sites was performed. A completion angiogram confirmed the excellent expansion of the stent graft with no evidence of types I, II, or III leaks (Figure 4).

Immediately following the procedure, the patient was unable to move his lower extremities. Back pain was noted as well, being higher than his baseline. He also complained of fecal and urinary incontinence. Upon examination, the patient had a 0/5 motor strength on bilateral hip flexion, knee flexion/extension, dorsiflexion, and plantarflexion. Deep tendon reflexes were absent in the lower extremities bilaterally. Plantar responses were muted bilaterally. The patient retained intact symmetrical sensations on light touch throughout, without a noted sensory level. The remainder of his physical examination was unremarkable. Neurology was consulted, and a norepinephrine infusion was initiated to maintain a mean arterial blood pressure of more than 100 mmHg. Immediate magnetic resonance imaging (MRI) did not show significant acute abnormalities. However, a lumbar drain was recommended and was placed by the neurosurgical team. There was an excellent return of the cerebrospinal fluid (CSF) under normal pressure. About 20 mL of CSF were drained every hour.

The patient’s neurological status gradually improved during his hospitalization. Shortly after lumbar drain placement, he was able to mildly move his hips and knees with a slight movement in his feet. A few days after his admission, he was able to move both lower extremities, although not against gravity. Fecal and urinary incontinence significantly improved. The lumbar drain was removed six days following its placement. By that time, examination revealed a 2/5 motor strength in bilateral hip flexors. The patient was able to adduct his hips, but with minimal abduction. Minimal dorsiflexion was noted on the right foot, but none on the left. He was able to minimally extend his first toe on the right side, not on the left. Deep tendon reflexes were absent in the lower extremities bilaterally. Sensations remained intact and symmetric to light touch throughout, without an evident sensory level.

**Figure 5:**
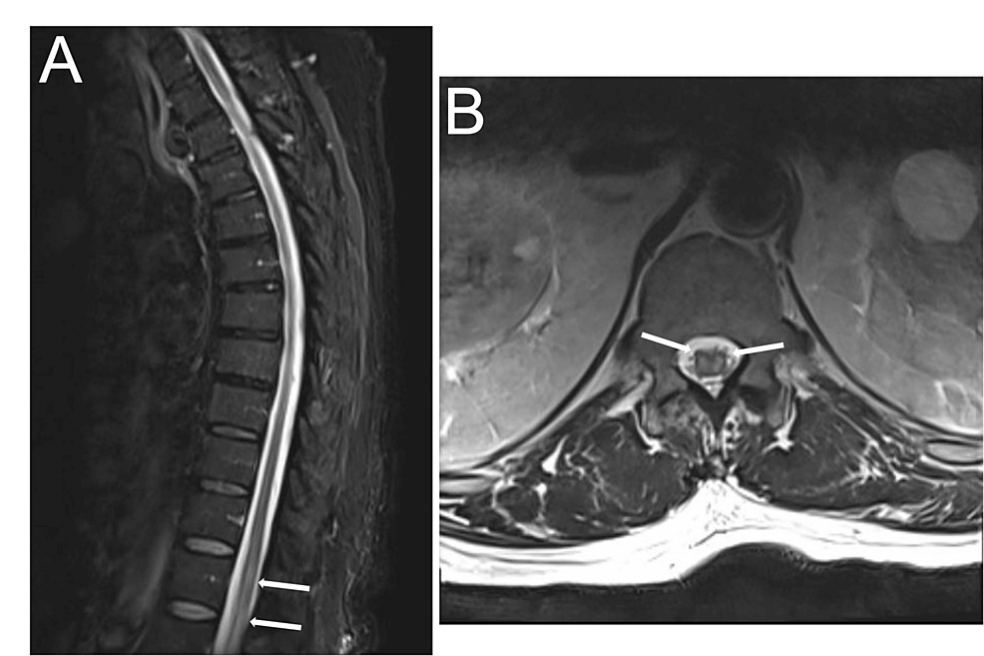
Follow-up MRI: magnetic resonance imaging sagittal (panel A) and axial (panel B) slices two weeks following discharge showing edema and expansion of the distal spinal cord with an abnormal signal on diffusion-weighted imaging concerning for ischemia of the conus medullaris (white arrows).

The patient was discharged to inpatient rehabilitation and subsequently returned home for physical rehabilitation. Follow-up MRI two weeks later showed edema and expansion of the distal spinal cord with an abnormal signal on diffusion-weighted imaging (DWI) concerning ischemia of the conus medullaris (Figure 5). Follow-up for one year showed improvement in the patient’s neurological status with physical and occupational therapy. He is able to ambulate for a few steps with the assistance of a walker. However, he remains predominantly wheelchair-dependent.

## Discussion

The differential diagnosis encompassed spinal cord ischemia, acute transverse myelopathy, acute viral myelitis, acute inflammatory demyelinating polyradiculopathy, and demyelinating diseases, including multiple sclerosis. Given the patient's acute manifestation following the EVAR procedure, her retained sensory functions, the lack of evidence or history of infections or inflammatory diseases, and the unremarkable MRI following the procedure, the patient was diagnosed with spinal cord ischemia.

SCI seems to be multifactorial in etiology. Predisposing factors include atheromatous embolization and interruption of collateral perfusion [3,8-10]. A systematic review by Moulakakis et al. reported comorbidities that included coronary artery disease, chronic obstructive pulmonary disease, diabetes mellitus, end-stage renal disease, peripheral arterial disease, chronic heart failure, and malignancy. Patients were reported to have had characteristics of technically challenging repairs, including angulation, reverse tapered necks, tortuosity of common iliac arteries, or calcified aortas [8,11]. In this case, the patient had a short left CIA.

In addition to its main feeding arteries, the spinal cord relies on a complex network of collaterals for its blood supply. These arise from the subclavian, vertebral, intercostal, lumbar, inferior mesenteric, internal iliac, and sacral arteries [12]. Deployment of the aortic stent graft commonly leads to an interrupted blood supply to the inferior mesenteric artery and all infrarenal lumbar branches and thus could jeopardize spinal perfusion. However, we agree with Kouvelos et al. that this could not exclusively explain the extremely low incidence of SCI following EVAR [13]. Given the coiling of the left IIA in our patient's case, a compromised right IIA could have possibly precipitated spinal cord ischemia. However, the preprocedural CTA and completion angiogram ruled out the occlusion of the right IIA. The artery of Adamkiewicz (AKA), also known as the great anterior radiculomedullary artery, supplies the anterior spinal artery, which in turn provides blood supply to the anterior two-thirds of the spinal cord. The AKA usually originates at the T8-L1 levels; hence, infrarenal stent grafts are less likely to occlude its orifice. However, variations in the AKA's origin are common [14]. Owing to this, the exclusion of the AKA by stent grafts has been reportedly implicated in SCI following EVAR procedures [11]. The AKA had not been identified in this patient, although the value of preprocedural assessment of the AKA's origin in preventing SCI remains uncertain. We believe that SCI in this patient was likely secondary to significant atherosclerosis/calcification of his iliac circulation.

There is no clear consensus on the management of EVAR-related SCI. Treatment has been predominantly based on case reports. In patients with suspected SCI, immediate imaging with CT or MRI is usually performed. Therapeutic modalities aim at enhancing spinal cord perfusion and reducing edema. These include increasing mean arterial blood pressure, cerebrospinal fluid drainage, intravenous steroids, naloxone, edaravone, hyperbaric oxygen, and therapeutic hypothermia [11,12,15]. Minimal to mild improvement in neurological functions is seen in most cases, although complete recovery has been reported [9,15,16].

Spinal cord protective measures during EVAR have been recommended by the U.S. Aortic Research Consortium, including maintaining a mean arterial pressure goal of not less than 90 mm Hg perioperatively, an intra- and post-operative hemoglobin level of not less than 10 mg/dL, and the use of prophylactic spinal drains in high-risk patients. Patients were classified as being at high risk if they required extended intended aortic length coverage, had previous open or endovascular aortic surgery, exhibited a "shaggy" atheromatous aorta, or had abnormal pelvic, bilateral vertebral, or left vertebral arterial perfusion [17].

An SCI risk stratification model for patients undergoing thoracic endovascular aortic repair has been proposed, while such a model is yet to exist for abdominal endovascular aortic repairs [18]. Early detection and prompt management could help prevent long-term adverse outcomes.

## Conclusions

SCI is a rare but devastating complication of EVAR. Symptoms most commonly present immediately following the procedure, although late presentations occur. The risk of SCI cannot be predicted prior to procedures; however, careful examination of pre-procedural CT scans could provide valuable information. Early recognition of this complication is of paramount importance in mitigating long-term sequelae. However, despite early management, most cases show no to minimal neurological improvement.
